# An Evaluation of the Hydrolytic Stability of Selected Experimental Dental Matrices and Composites

**DOI:** 10.3390/ma15145055

**Published:** 2022-07-20

**Authors:** Agata Szczesio-Wlodarczyk, Karolina Kopacz, Malgorzata Iwona Szynkowska-Jozwik, Jerzy Sokolowski, Kinga Bociong

**Affiliations:** 1 University Laboratory of Materials Research, Medical University of Lodz, Pomorska 251, 92-213 Lodz, Poland; 2 “DynamoLab” Academic Laboratory of Movement and Human Physical Performance, Medical University of Lodz, ul. Pomorska 251, 92-216 Lodz, Poland; karolina.kopacz@umed.lodz.pl; 3 Department of Health Sciences, Medical University of Mazovia, Ludwika Rydygiera 8, 01-793 Warszawa, Poland; 4 Faculty of Chemistry, Institute of General and Ecological Chemistry, Lodz University of Technology, Zeromskiego 116, 90-543 Lodz, Poland; malgorzata.szynkowska@p.lodz.pl; 5 Department of General Dentistry, Medical University of Lodz, ul. Pomorska 251, 92-213 Lodz, Poland; jerzy.sokolowski@umed.lodz.pl (J.S.); kinga.bociong@umed.lodz.pl (K.B.)

**Keywords:** neat resin, composites, hydrolytic stability, aging, clinical performance

## Abstract

Materials with potential use as dental restoration should be evaluated in an aggressive environment. Such accelerated aging is widely used in other industries and allows the assessment of service life. In the presented study, three neat resins (UDMA/Bis-GMA/TEGDMA 70/10/20 wt.%, UDMA/Bis-GMA/TEGDMA 40/40/20 wt.% and UDMA/Bis-EMA/TEGDMA 40/40/20 wt.%) and three composites based on these matrices were tested before and after aging protocols (I-7500 cycles, 5 °C and 55 °C, water and 7 days, 60 °C, 0.1 M NaOH; II-5 days, 55 °C, water and 7 days, 60 °C, 0.1 M NaOH). Flexural strength (FS), diametral tensile strength (DTS) and hardness (HV) were determined. Applied aging protocols resulted in a decrease in the value of the FS, DTS and HV. Larger changes were noticed for the neat resins. Materials in which the content of bis-GMA was lower or substituted by bis-EMA showed better resistance to degradation. The choice of mixtures with monomers characterized by lower sorption values may favorably affect hydrolytic stability. It was shown that for composites there was a drastic decrease in hardness, which suggests a more superficial effect of the used protocols. However, degradation of the surface layer can result in a growing problem over time given that the mastication processes are an inherent element in the oral environment.

## 1. Introduction

A dental composite consists of a polymer matrix, coupling agent and fillers, together with initiators and inhibitors of polymerization, UV stabilizers, dyes and various other additives. Each of these ingredients will in some way shape the final properties of the tooth reconstruction [1]. Most currently-available dental materials are based on conventional resins such as bisphenol A–glycidyl dimethacrylate (Bis-GMA), triethylene glycol dimethacrylate (TEGDMA), urethane dimethacrylate (UDMA) and ethoxylated bisphenol–A dimethacrylate (Bis-EMA) [2,3]. The final properties result from the interactions that arise in the monomer mixture and the quality of the resulting polymer network, not only from the characteristics of individual monomers [4]. Previous research has mainly focused on composites with two monomers; however, due to possible interactions between monomers, more complex systems also demonstrate good properties, especially when composites contain an equal mixture of Bis-GMA, Bis-EMA and UDMA [5,6,7,8].

The first step in creating a new dental composite should be the selection of an appropriate polymer matrix and filler, and this should be followed by a determination of the hydrolytic resistance of the material. The oral environment is continuously changing and very complex, hence it influences the clinical performance of dental materials [9]. Ageing of the resin composites results in the deterioration of the structure and mechanical properties of dental material. Unfortunately, no requirements for clinical performance or lifetime prediction are needed for new dental materials introduced to the market. The only test that can partially assist with material evaluation is the determination of water sorption and solubility based on ISO standard (4049:2019 Dentistry—Polymer-based restorative materials) [10]. High values of those characteristics are undesirable as they may weaken the restoration [11]. However, these studies do not provide information on how the material will behave during prolonged use in a complex oral environment. Recent studies made the first step in standardizing the aging protocol, which will be useful for evaluating dental composites. The tested protocols caused degradation of the polymer matrix as well as the filler and the matrix–filler interface. The more complex protocols based on both thermal and chemical ageing were proposed to be more accurate in the evaluation of composites’ long term clinical performance [12].

The present study examines three matrices and composites based on these resins. All materials were subjected to two ageing protocols proposed in a previous study [12]. The aim of the research was to determine the hydrolytic stability of selected materials (resins and composites), as well as their flexural strength, diametral tensile strength and hardness. The null hypothesis was that the properties of the resins and composites remain stable following a complex aging protocol.

## 2. Materials and Methods

Three different resin mixtures were prepared according to the weight percentage of selected monomers. Bis-GMA, UDMA, TEGDMA and Bis-EMA were delivered by Esstech Inc. (Essington, PA, USA). Each mixture contained camphorquinone (<1 wt.%) and N,N-dimethylaminoethyl methacrylate. UDMA/Bis-GMA/TEGDMA 70/10/20 wt.%, 40/40/20 wt.% and UDMA/Bis-EMA/TEGDMA 40/40/20 wt.% matrices were tested. The resins were also evaluated after filling with 45 wt.% of silica (Arsil, Zakłady Chemiczne “RUDNIKI” S.A., Rudniki, Poland) silanized with γ-Methacryloxypropyltrimethoxy silane (Unisil Sp. z o. o., Tarnów, Poland). Silicone molds were used to prepare samples. In order to avoid the formation of an inhibition layer, the surfaces of the materials were covered with a polyester tape (Hawe Striproll, Kerr, Bioggo, Switzerland) and the glass slides. The materials were cured for 20 s for 2 mm thickness. Specimens were polymerized using an LED curing light (the CURE—TC-01, Spring Health Products, Norristown, PA, USA) with direct contact of optical fiber with the glass surface. The lamp has 1250 mW/cm^2^ light irradiance. The composition of the tested materials is given in Table 1.

The impact of selected aging protocols (Table 2) on flexural strength, diametral tensile strength and hardness were determined. The protocols were selected on the basis of previous research [12]. The combination of thermal and chemical factors may better mimic the prolonged use of composite materials in the oral cavity. Long-term performance of dental composites in the oral environment can be evaluated using accelerated aging with more aggressive/increased factors. The use of the NaOH solution significantly accelerates the hydrolytic degradation, while the increased temperature also allows for the acceleration of the sorption and dissolution processes. The use of thermocycles (7500 cycles, 5 °C and 55 °C) had a greater effect than aging in water (5 days, 55 °C), especially on the material with a higher filler content. However, the protocol can provide an alternative for studies without specialized thermocycling equipment. Taking this into account, and knowing that such a prediction is complex, it can be assumed that the proposed aging protocols will simulate several years in the oral environment [12].

In aging solutions distilled water was used, which was obtained with the Millipore Direct-Q 5 UV purification system (Millipore SAS, Molsheim, France). The samples in plastic dishes were placed in an incubator (DZ-2BCII Vacuum Drying Oven, ChemLand, Stargard Szczeciński, Poland) at the specified temperature and times (Table 2). To prepare the NaOH solution, the determined amount of substance (Avantor Performance Materials, Poland, Gliwice) was measured on an analytical balance (Radwag XA 82/220/X, Puszczykowo, Poland), and the solution was further prepared in a volumetric flask. The thermocycling machine (THE 1200, SD Mechatronic, Feldkirchen-Westerham, Germany) was used to perform 7500 thermocycles in water for 20 s of dwell time at 5 and 55 °C.

Flexural strength (FS) was established based on ISO 4049:2019 [10]. Seven measurements using rectangular samples (25 mm long, 2 mm wide, 2 mm thick) were made for each study group. The tensile properties of brittle materials, such as dental composites, are commonly determined using diametral tensile strength (DTS) [13,14,15]. The DTS measurements were made on nine cylindrical samples (diameter 6 mm and thickness 3 mm) for each study group. The mechanical properties (FS, DTS) were determined using a Zwick Roell Z020 universal testing machine (Zwick–Roell, Ulm, Germany). The traverse speed was 2 mm/min in DTS test and 1 mm/min in FS. The hardness of the tested materials was measured using the Vickers method (1000 g applied load, 10 s penetration time). Nine measurements were performed for each study group. The Zwick ZHV2–m hardness tester (Zwick–Roell, Ulm, Germany) was used for the tests. The methods are extensively described in a previous manuscript [8]. In addition, surface free energy (SFE) of composites’ was determined using contact angle measurements. The pair of non-polar and polar liquid (water and diiodomethane) was used. Five droplets of each liquid were made for tested composite materials. The Owens and Wendt approach was used to calculate the *SFE* (Equations (1)–(3)). SFE is the sum of the dispersion (*γ^d^*) and polar components (*γ^p^*).

(1) $$SFE=\gamma^{d}+\gamma^{p}$$

(2) $$\gamma^{d}={\left(\frac{\left(\sqrt{\gamma_{1}^{p}}\cdot \gamma_{2}\cdot \left(\cos\theta_{2}+1\right)\right)-\left(\sqrt{\gamma_{2}^{p}}\cdot \gamma_{1}\cdot \left(\cos\theta_{1}+1\right)\right)\;}{2\cdot \left(\sqrt{\gamma_{2}^{d}\cdot \gamma_{1}^{p}}-\sqrt{\gamma_{2}^{p}\cdot \gamma_{1}^{d}}\right)}\right)}^{2}$$

(3) $$\gamma^{p}={\left(\frac{\left(\gamma_{1}\cdot \left(\cos\theta_{1}+1\right)\right)-\left(2\cdot \sqrt{\gamma_{1}^{d}\cdot \gamma^{d}}\right)\;}{2\cdot \left(\sqrt{\gamma_{1}^{p}}\right)}\right)}^{2},$$

where: *γ_d_* is the dispersion component of SEF, *γ^p^* is the polar component of SEF, $\gamma_{1}^{d}$ is the dispersion component of water (21.6 mJ/m^2^), $\gamma_{1}^{p}$ is the polar component of water (51.0 mJ/m^2^), γ_1_ is the sum of the dispersion and polar components of water (72.6 mJ/m^2^), *θ*_1_ is the water contact angle, $\gamma_{2}^{d}$ is the dispersion component of diiodomethane (48.5 mJ/m^2^), $\gamma_{2}^{p}$ is the polar component of diiodomethane (2.3 mJ/m^2^), *γ*_2_ is the sum of dispersion and polar components of diiodomethane (72.6 mJ/m^2^), and *θ*_2_ is the diiodomethane contact angle.

Statistica version 13 software (StatSoft, Kraków, Poland) was used for statistical analysis. The distribution of continuous variables was tested using the Shapiro–Wilk test of normality. Based on the results, the Kruskal–Wallis test with multiple comparisons of mean ranks or the one-way ANOVA with post hoc test (Fisher’s least significant difference) were applied. The accepted level of significance was α = 0.05. Based on their distribution and homogeneity of variance, the results are given as either mean values with standard deviation (SD) or median values with quartile deviation (QT).

## 3. Results

The values are presented as mean with standard deviation for variables with normal distribution and homogeneity of variance, or median with quartile deviation for non-normally distributed variables. The obtained results of neat resin are presented in Table 3.

The applied aging protocols, compared to the control group, had a significant impact on the tested properties of the three resins.

The results of the evaluation of composites materials are presented in Table 4.

**Figure A1:**
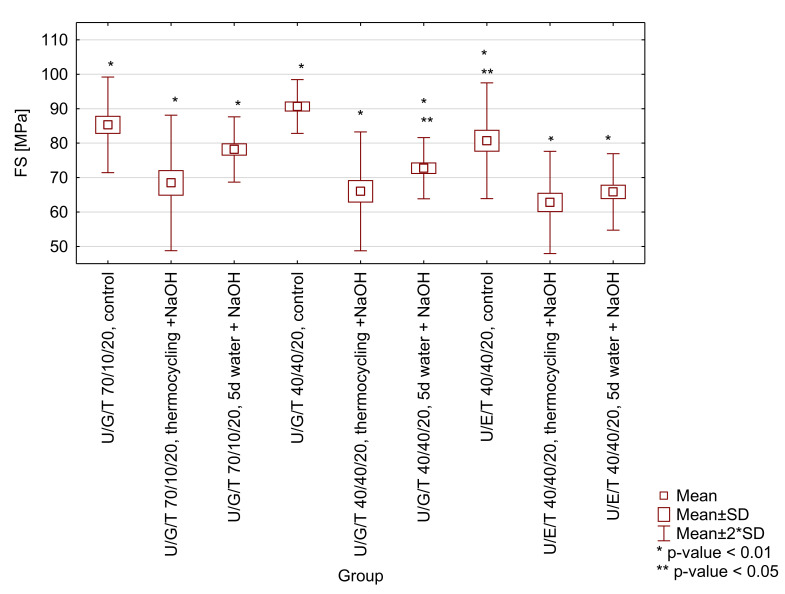
Box-and-whisker plot of three-point bending flexural strength (FS) of tested matrices (UDMA/Bis-GMA/TEGDMA 70/10/20 wt.% and 40/40/20 wt.% and UDMA/Bis-EMA/TEGDMA 40/40/20 wt.%) after selected ageing protocols (control-water, 24 h, 37 °C; thermocycling + NaOH-water, 7500 thermo cycles, 5/55 °C and 0.1 M NaOH, 7 days, 60 °C; 5 d water + NaOH-water, 5 days, 55 °C and 0.1 M NaOH, 7 days). Significant differences occur in a given group with a probability of *p* < 0.01 (*) and/or *p* < 0.05 (**).

**Figure A2:**
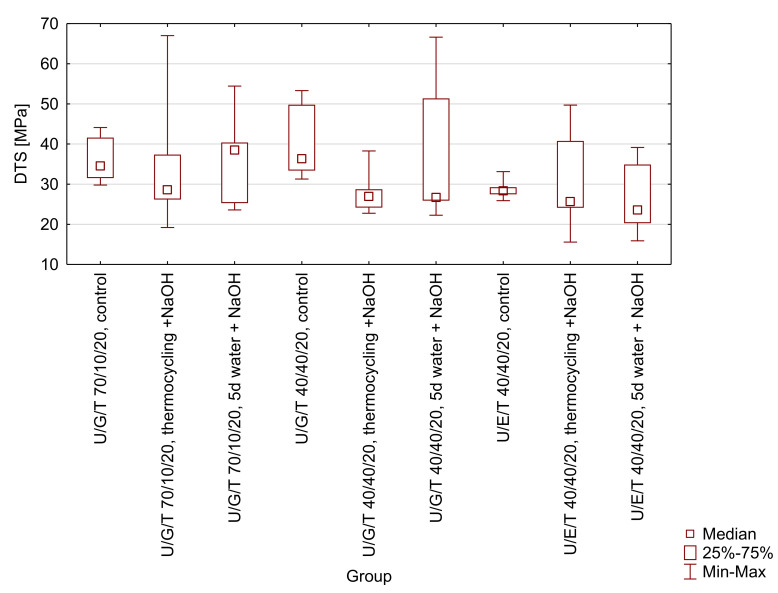
Box-and-whisker plot of diametral tensile strength (DTS) of tested matrices (UDMA/Bis-GMA/TEGDMA 70/10/20 wt.% and 40/40/20 wt.% and UDMA/Bis-EMA/TEGDMA 40/40/20 wt.%) after selected ageing protocols (control-water, 24 h, 37 °C; thermocycling + NaOH-water, 7500 thermo cycles, 5/55 °C and 0.1 M NaOH, 7 days, 60 °C; 5d water + NaOH-water, 5 days, 55 °C and 0.1 M NaOH, 7 days). No significant differences were observed.

**Figure A3:**
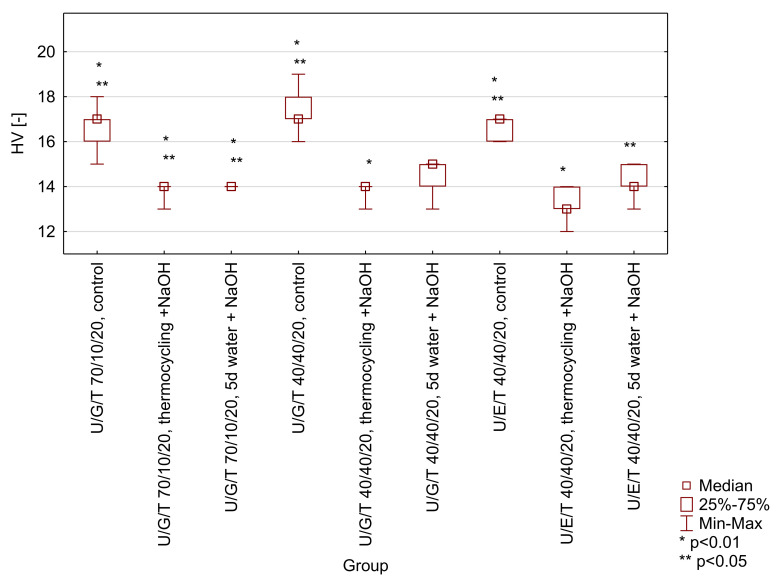
Box-and-whisker plot of Vickers hardness (HV) of tested matrices (UDMA/Bis-GMA/TEGDMA 70/10/20 wt.% and 40/40/20 wt.% and UDMA/Bis-EMA/TEGDMA 40/40/20 wt.%) after selected ageing protocols (control-water, 24 h, 37 °C; thermocycling + NaOH-water, 7500 thermo cycles, 5/55 °C and 0.1 M NaOH, 7 days, 60 °C; 5 d water + NaOH-water, 5 days, 55 °C and 0.1 M NaOH, 7 days). Significant differences occur in a given group with a probability of *p* < 0.01 (*) and/or *p* < 0.05 (**).

**Figure A4:**
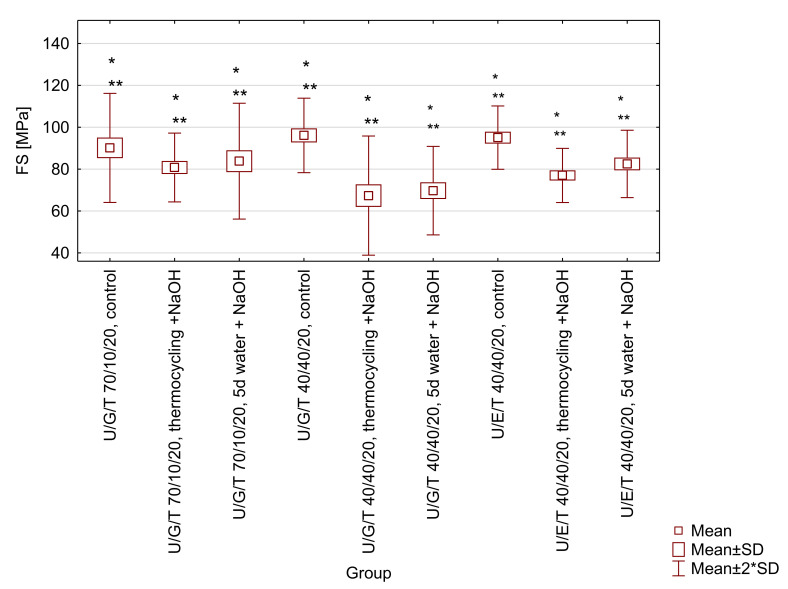
Box-and-whisker plot of three-point bending flexural strength (FS) of tested composites (UDMA/Bis-GMA/TEGDMA 70/10/20 wt.% and 40/40/20 wt.% and UDMA/Bis-EMA/TEGDMA 40/40/20 wt.%) after selected aging protocols (control-water, 24 h, 37 °C; thermocycling + NaOH-water, 7500 thermo cycles, 5/55 °C and 0.1 M NaOH, 7 days, 60 °C; 5d water + NaOH-water, 5 days, 55 °C and 0.1 M NaOH, 7 days). Significant differences occur in a given group with a probability of *p* < 0.01 (*) and/or *p* < 0.05 (**).

**Figure A5:**
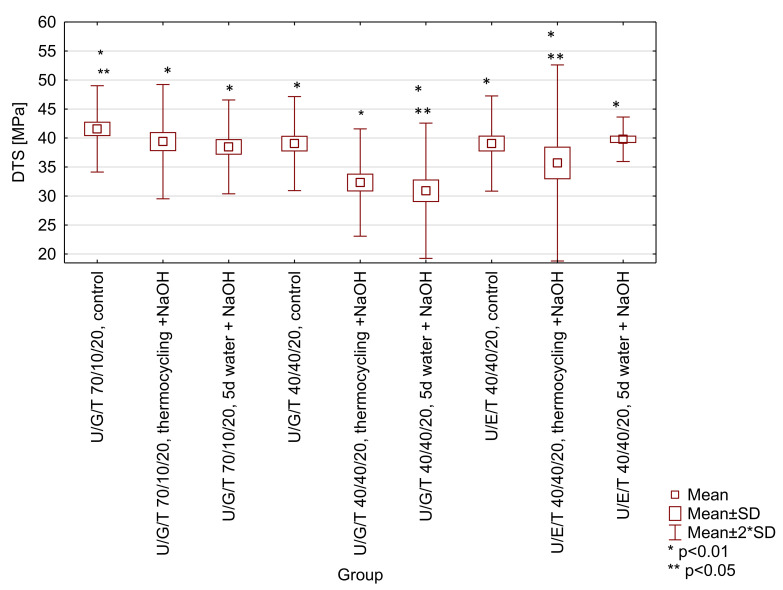
Box-and-whisker plot of diametral tensile strength (DTS) of tested composites (UDMA/Bis-GMA/TEGDMA 70/10/20 wt.% and 40/40/20 wt.% and UDMA/Bis-EMA/TEGDMA 40/40/20 wt.%) after selected ageing protocols (control-water, 24 h, 37 °C; thermocycling + NaOH-water, 7500 thermo cycles, 5/55 °C and 0.1 M NaOH, 7 days, 60 °C; 5d water + NaOH-water, 5 days, 55 °C and 0.1 M NaOH, 7 days). Significant differences occur in a given group with a probability of *p* < 0.01 (*) and/or *p* < 0.05 (**).

**Figure A6:**
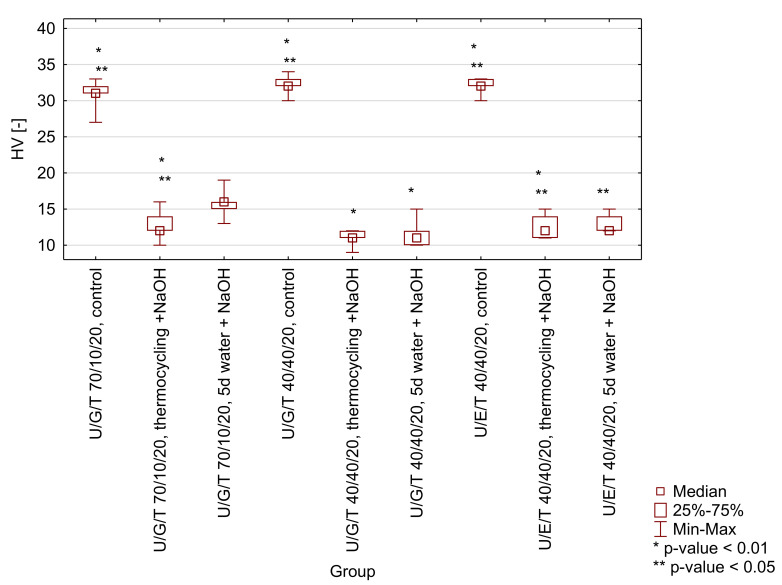
Box-and-whisker plot of Vickers hardness (HV) of tested composites (UDMA/Bis-GMA/TEGDMA 70/10/20 wt.% and 40/40/20 wt.% and UDMA/Bis-EMA/TEGDMA 40/40/20 wt.%) after selected ageing protocols (control-water, 24 h, 37 °C; thermocycling + NaOH-water, 7500 thermo cycles, 5/55 °C and 0.1 M NaOH, 7 days, 60 °C; 5d water + NaOH-water, 5 days, 55 °C and 0.1 M NaOH, 7 days). Statistical differences occur in a given group with a probability of *p* < 0.01 (*) and/or *p* < 0.05 (**).

Box-and-whisker plots of the collected results and exact *p*-values are provided in the Appendix A (Figure A1, Figure A2, Figure A3, Figure A4, Figure A5 and Figure A6 and Table A1, Table A2, Table A3, Table A4 and Table A5).

The result of surface free energy analysis by contact angle measurements of the pair of non-polar and polar liquid (water and diiodomethane) are presented in Table 5. Additional data (dispersion and polar component) are provided in the Appendix A (Table A6).

## 4. Discussion

All composite materials based on a polymer matrix will be subject to aging processes during their use, which will affect their operating life. The environment in which the product is used plays a key role in its behavior over time [16,17]. Dental composites that are used in the oral cavity are exposed to a very complex and often changing environment. However, the tests used to evaluate these materials do not typically include research on accelerated aging with increased environmental factors [9], even though such studies are widely used in other industries [17]. Our findings based on analyses of the hydrolytic resistance of selected matrices and composites indicate that the properties of the resins and composites are not stable following complex aging protocols. Therefore, the null hypothesis can be rejected.

There are three important elements of the dental composite that can degrade during aging: resin, filler and coupling agent [18,19]. This degradation usually takes place through hydrolytic processes that can be accelerated by environmental conditions and factors such as enzymes [20]. The tested materials were found to undergo deterioration over time, and this finding is consistent with other studies, which have also noted poorer mechanical strength, hardness and abrasion resistance over time [9,21,22]. Aging media diffuse through the material and lead to plasticization, softening and swelling of the polymer matrix [23,24]. Chemical degradation of the polymer matrix, filler and coupling agent occurs. These physical and chemical changes result in unreacted monomers, degradation products and filler particles being eluted, resulting in the formation of voids and cracks and the weakening of the polymer matrix structure, and the eventual deterioration of material properties [18].

Aging in water was not associated with any great reduction in the properties of dental composites; however, the results depended mainly on the length of storage in water [25,26,27]. Taking into account a complex oral environment, aging protocols that use water alone are insufficient to predict the clinical performance of biomaterials. As resin-based dental composites are hydrolyzed by interaction with an OH^−^ ion, alkaline mediums with a high content of hydroxyl ions can be used as aging media in in vitro testing. NaOH solution is commonly used to accelerate aging media in dental composite testing [28]. This medium has been shown to cause structural damage in the subsurface layer, and to induce microscopic cracks, reduce mechanical properties and degrade the interface, resulting in the filler debonding [9,29,30,31]. The thermocycling protocol had a greater impact on the tested materials. As thermal cycles can occur in the oral cavity from 20 to 50 times a day [32,33], it seems appropriate to use thermocycles to evaluate the operating life of dental composites. The deterioration effect has been attributed to differences in thermal conductivity between resin matrix and filler. This can lead to greater plasticization and swelling of the polymer matrix, which will be more susceptible to the penetration of NaOH, which acts as an aggressive aging medium. Our results indicate that this complex method accelerates aging processes in tested material and can be useful to evaluate clinical performance in vitro.

It was noticed that tested materials demonstrate different degrees of their observed changes. In this case, as all tested fillers used the same coupling agent and filler, their distinguishing feature is the composition of the polymer matrix. The monomer type and content of a filler determines its material properties, such as water sorption. The chemical hydrolysis of polymer matrix is caused by the presence of water or aging media in dental composites. High water sorption values can thus promote degradation [29,34]. The tested materials are based on conventional monomers such as UDMA, TEGDMA, Bis-GMA and Bis-EMA. The UDMA and Bis-GMA monomers include urethanes and hydroxyl functional groups, which can bind water molecules [35], while the ethylene oxide linkages in the TEGDMA monomer can easily accept hydrogen bonds. The presence of two phenyl rings creates a spacious barrier around ethoxylated groups, making the Bis-EMA monomer more hydrophobic [35]. The water sorption values of the monomers used in this study are 51.2 µg/mm^3^ for Bis-GMA, 42.3 µg/mm^3^ for UDMA, 28.8 µg/mm^3^ for TEGDMA and 21.3 µg/mm^3^ for Bis-EMA [36]. From the results obtained during the SFE analysis (Table 5), it can be concluded that all composite materials are hydrophilic due to their water contact angle being between 10° and 90° [37]. Composites with bis-GMA have similar values (UDMA/bis-GMA/TEGDMA 70/10/20 and UDMA/bis-GMA/TEGDMA 40/40/20), but the composite with a higher content of bis-GMA is slightly more on hydrophilic side. The material containing bis-EMA (UDMA/bis-EMA/TEGDMA 40/40/20) is significantly different and it has a more hydrophobic character. The obtained results confirm that the content of bis-GMA increases the hydrophilic character of dental materials [38]. Surface Free Energy (SFE) values are similar for all tested materials. Although the method of testing wettability and SFE is relatively simple to perform, it only characterized surface properties, which are different to the bulk properties [39]. SFE and wettability is a more important aspect in adhesion and bonding. Water sorption or water absorbency studies may better evaluate the material and its behavior in the oral cavity (aquatic environment) over time [40,41,42]. Water absorbency (Ab) and dissolution (Ds) after 120 days of neat resins were investigated previously [8]. The highest values were observed for UDMA/bis-GMA/TEGDMA 70/10/20 (Ab: 3.0348 ± 0.1340 wt.% and Ds: 0.6208 ± 0.1615 wt.%). The lowest values were obtained for UDMA/bis-EMA/TEGDMA 40/40/20 matrix (Ab: 1.8683 ± 0.0778 wt.% and Ds: 0.3243 ± 0.0796 wt.%). Neat resin UDMA/bis-GMA/TEGDMA 40/40/20 has an absorbency of 2.8182 ± 0.1324 wt.% and a dissolution of 0.6192 ± 0.0604 wt.%. In addition, taking into account that the filler phase and silane coupling agent were the same for all composites, it can be assumed that materials with more Bis-GMA will have a higher water sorption value, and that materials based on the UDMA/Bis-GMA/TEGDMA 40/40/20 wt.% matrix show a more significant deterioration of properties during the aging procedures (Table 3 and Table 4).

It remains undecided which composite component is primarily responsible for degradation. In our study, the matrices themselves demonstrated a greater reduction in strength properties (FS, DTS) than for the corresponding composite materials containing filler. Similar observations were noted in a previous study dealing with the establishment of aging protocols for composite materials [12]. A study of composite fillers stored in ethanol solution found that the tested composites lost fracture toughness, which the authors attribute to a weakening of the resin matrix [43]. In another study, high filled composites were found to be more resistant to aging, suggesting that the quantity of artificial aging may depend on filler content [30]. It can be explained that when no filler is present, greater volumes of aging media are able to penetrate to the polymer network, thus significantly influencing the degradation rate of the material. Typically, composites demonstrate similar responses to aging protocols as unfilled matrices; this may be due to small amount of filler content, which may only be 45 wt.%. Therefore, it may be possible to improve hydrolytical degradation by obtaining highly-filled composites.

Chemical or thermal aging change the microstructure of the dental composite [19,44,45,46]. It was shown that after conditioning in 0.1 M NaOH for 7 days at 60 °C or using 7500 thermo cycles, 5/55 °C, filler particles seem to have been debonded and plucked out. In addition, macrofilles demonstrate an additional fracture. The surface of the neat resin showed some ongoing degradation–delamination, and peeling [12].

The greatest changes were observed in the hardness of all tested composite materials. For most of the studied groups, the values after aging were more than 60% lower than those of the controls. No such changes were observed for polymer matrices without filler. Similar results were obtained by Cilli et al. [47]. It is believed that water penetrates not only through the polymer matrix but also through the interface between filler particle and the organic matrix, resulting in the degradation of the silane coupling agent and fillers [48,49]. The alkaline medium used as an aging medium provides many more hydroxyl ions than water alone [50]; these break the siloxane bonds in the silane coupling agent, resulting in the degradation of the silica network and surface, thus reducing the hardness of the material [51]. The effect, however, is superficial; the strength results do not indicate a large propagation of degradation into the bulk of the material. However, taking into account the continuous friction applied by chewing forces in the oral environment, it can be assumed that the top layer will be successively lost, revealing subsequent parts of the material and allowing the restoration to be continually eroded [52]. In addition, continuous hydrolysis of the coupling agent can open up an extra pathway for aging media to diffuse, which in turn may accelerate the degradation of a restoration [48,53,54]. Nevertheless, it has to be remembered that, in clinical practice, finishing and polishing procedures are generally performed, which may have influence on the abrasion mechanism in the oral environment [55].

Taking into account that, during clinical use, dental materials are affected by a range of factors which may accelerate the aging processes, they should be subjected to complex evaluation procedures. Our findings indicate that the examined properties decrease following complex aging protocols, with the degree of change depending on the matrix composition. The matrix containing the greatest amount of Bis-GMA (40 wt.%) monomer was found to be less hydrolytically stable than the other tested materials due to its high water sorption values. For this reason, a composite containing Bis-EMA can be a better choice. In addition, considering the concerns about the harmfulness of the Bis-GMA monomer, a composite based on UDMA/bis-EMA/TEGDMA 40/40/20 seems to be a good choice for future research. An interesting aspect to investigate in the future would be the influence of the degree of filling on hydrolytic stability.

## 5. Conclusions

These studies took into account only three selected neat resins and composites based on these matrices; the analysis included research methods popular in dentistry research. In order to better understand the problem and confirm our conclusions, research should be carried out on a wider group of materials and more testing methods should be provided.

On the basis of the obtained results and with the above mentioned limitations of study in mind, the following conclusions can be drawn:

1. The use of aging protocols can help evaluate materials for clinical use.
2. One of the main factors responsible for changes in the strength properties during aging is the polymer matrix and the polymer matrix–filler interface.
3. In composites, the changes induced by the protocols used have a significant impact on the surface layer, which can cause a growing problem over time.
4. The choice of mixtures with monomers characterized by lower sorption values may favorably affect hydrolytic stability.

Standardization of the aging protocol for dental materials is a separate project (funded by National Science Centre, Poland grant number: UMO-2020/37/N/ST5/00191, 2021). The research included in this project will also evaluate a wide range of different types of composite materials. The results of preparing studies may indicate which components of composite materials allow us to achieve a clinically stable material for the reconstruction of lost tooth tissues.

## Figures and Tables

**Table 1:** Composition of tested matrices. Composites used in this study have the same resin compositions and contain 45 wt.% of silanized silica.

Material Signature	UDMA Content (wt.%)	TEGDMA Content (wt.%)	Bis-GMA Content (wt.%)	Bis-EMA Content (wt.%)
UDMA/bis-GMA/TEGDMA 70/10/20	70	20	10	-
UDMA/bis-GMA/TEGDMA 40/40/20	40	20	40	-
UDMA/bis-EMA/TEGDMA 40/40/20	40	20	-	40

**Table 2:** Description of selected ageing protocols used to evaluate tested materials.

Ageing Protocol	Description
control	24 h, 37 °C, distilled water
thermocycling + NaOH	7500 cycles, 5 °C and 55 °C, water and 7 days, 60 °C, 0.1 M NaOH
5d water + NaOH	5 days, 55 °C, water and 7 days, 60 °C, 0.1 M NaOH

**Table 3:** The flexural strength (FS), diametral tensile strength (DTS) and hardness (HV) of tested matrices after selected ageing protocols. Normally-distributed variables are given as means with standard deviation, while non-normally distributed variables are given as median with quartile deviation. The results with the same assigned letter show a significant difference at the level of *p* ≤ 0.05.

Matrix	Ageing Protocol	FS [MPa]	SD	DTS [MPa]	QT	HV [-]	QT
UDMA/bis-GMA/TEGDMA 70/10/20	Control	85.3 ^a,b,c,d,e^	6.9	34.5	10.1	17 ^a,b,c,d^	1
	thermocycling + NaOH	68.5 ^a,f,m,r^	9.8	28.6	11.2	14 ^a,e,j^	0
	5 d water + NaOH	78.2 ^g,r,s,t,w^	4.7	38.5	15.1	14 ^b,f,k^	0
UDMA/bis-GMA/TEGDMA 40/40/20	Control	90.6 ^f,g,h,i,j,k,l^	3.9	36.3	16.4	17 ^e,f,g,h,i^	1
	thermocycling + NaOH	66 ^b,h,n,s^	8.6	27	4.6	14 ^c,g,l^	0
	5 d water + NaOH	72.7 ^c,i,o,y^	4.4	26.7	25.5	15	1
UDMA/bis-EMA/TEGDMA 40/40/20	Control	80.7 ^j,m,n,o,p,q^	8.4	28.3	1.8	17 ^j,k,l,m^	1
	thermocycling + NaOH	62.8 ^d,k,p,t,y^	7.4	25.7	16.7	13 ^d,h,m^	1
	5 d water + NaOH	65.8 ^e,l,q,w^	5.6	23.6	14.6	14 ^i^	1

Ageing protocols: control-water, 24 h, 37 °C; thermocycling + NaOH-water, 7500 thermo cycles, 5/55 °C and 0.1 M NaOH, 7 days, 60 °C; 5 d water + NaOH-water, 5 days, 55 °C and 0.1 M NaOH, 7 days.

**Table 4:** The flexural strength (FS), diametral tensile strength (DTS) and hardness (HV) of the tested composites after selected ageing protocols. Normally-distributed variables are given as means with standard deviation, while non-normally distributed variables are given as median with quartile deviation. The results with the same assigned letter show a significant difference at the level of *p* ≤ 0.05.

Composite	Ageing Protocol	FS [MPa]	SD	DTS [MPa]	SD	HV [-]	QT
UDMA/bis-GMA/TEGDMA 70/10/20	Control	90.1 ^g,l,u^	13.0	41.6 ^a,g,n^	3.7	31 ^a,b,c,d^	1
	thermocycling + NaOH	80.8 ^a,h,p^	8.2	39.4 ^b,h^	4.9	12 ^a,e,i^	2
	5 d water + NaOH	83.8 ^b,i,m,r^	13.8	38.5 ^c,i^	4.0	16	1
UDMA/bis-GMA/TEGDMA 40/40/20	Control	96.1 ^a,b,c,d,e,f^	8.9	39.0 ^d,j^	4.1	32 ^e,f,g,h,i^	1
	thermocycling + NaOH	67.3 ^c,g,h,i,j,k^	14.2	32.3 ^a,b,c,d,e,f^	4.6	11 ^b,f,k^	1
	5 d water + NaOH	69.7 ^d,l,m,n,o^	10.6	30.9 ^g,h,i,j,k,l,m^	5.8	11 ^c,g,l^	2
UDMA/bis-EMA/TEGDMA 40/40/20	Control	95.0 ^j,n,p,r,s,t^	7.6	39.0 ^e,k^	4.1	32 ^j,k,l,m,n^	1
	thermocycling + NaOH	77.0 ^e,s,u^	6.5	35.7 ^l,n^	8.5	12 ^d,h,m^	3
	5 d water + NaOH	82.5 ^f,k,o,t^	8.1	39.8 ^f,m^	1.9	12 ^i,n^	2

Ageing protocols: control-water, 24 h, 37 °C; thermocycling + NaOH-water, 7500 thermo cycles, 5/55 °C and 0.1 M NaOH, 7 days, 60 °C; 5 d water + NaOH-water, 5 days, 55 °C and 0.1 M NaOH, 7 days.

**Table 5:** Water and diiodomethane contact angle and surface free energy (SFE) of tested composites.

Composite	Water Contact Angle [°]	SD	Diiodomethane Contact Angle [°]	SD	SFE [mJ/m^2^]	SD
UDMA/bis-GMA/TEGDMA 70/10/20	70	4	42	8	42	3
UDMA/bis-GMA/TEGDMA 40/40/20	74	2	38	4	42	1
UDMA/bis-EMA/TEGDMA 40/40/20	82	3	27	6	45	2

## Data Availability

Data available in a publicly-accessible repository: Zenodo at: 10.5281/zenodo.6865424.

## Appendix A

**Table A1:** Probabilities for *post hoc* testing for three-point bending flexural strength (FS) of tested matrices. Tested group: 1-UDMA/bis-GMA/TEGDMA 70/10/20, control; 2-UDMA/bis-GMA/TEGDMA 70/10/20, thermocycling +NaOH, 3-UDMA/bis-GMA/TEGDMA 70/10/20, 5 d water + NaOH, 4-UDMA/bis-GMA/TEGDMA 40/40/20, control; 5-UDMA/bis-GMA/TEGDMA 40/40/20, thermocycling +NaOH; 6-UDMA/bis-GMA/TEGDMA 40/40/20, 5d water + NaOH; 7-UDMA/bis-EMA/TEGDMA 40/40/20, control; 8-UDMA/bis-EMA/TEGDMA 40/40/20, thermocycling +NaOH, 9-UDMA/bis-EMA/TEGDMA 40/40/20, 5 d water + NaOH.

	1	2	3	4	5	6	7	8	9
1	-	0.000031	0.059365	0.156527	0.000003	0.001298	0.218781	0.000000	0.000003
2		-	0.011409	0.000000	0.512874	0.254477	0.001710	0.132024	0.483872
3			-	0.001424	0.001831	0.147992	0.498258	0.000119	0.001597
4				-	0.000000	0.000012	0.009712	0.000000	0.000000
5					-	0.075780	0.000221	0.387831	0.963301
6						-	0.036092	0.009712	0.068805
7							-	0.000012	0.000190
8								-	0.413359
9									-

Ageing protocols: control–water, 24 h, 37 °C; thermocycling + NaOH–water, 7500 thermo cycles, 5/55 °C and 0.1 M NaOH, 7 days, 60 °C; 5 d water + NaOH-water, 5 days, 55 °C and 0.1 M NaOH, 7 days, 60 °C.

**Table A2:** Probabilities for multiple comparison testing for Vickers hardness (HV) of tested matrices. Tested group: 1-UDMA/bis-GMA/TEGDMA 70/10/20, control; 2-UDMA/bis-GMA/TEGDMA 70/10/20, thermocycling +NaOH, 3-UDMA/bis-GMA/TEGDMA 70/10/20, 5d water + NaOH, 4-UDMA/bis-GMA/TEGDMA 40/40/20, control; 5-UDMA/bis-GMA/TEGDMA 40/40/20, thermocycling +NaOH; 6-UDMA/bis-GMA/TEGDMA 40/40/20, 5d water + NaOH; 7-UDMA/bis-EMA/TEGDMA 40/40/20, control; 8-UDMA/bis-EMA/TEGDMA 40/40/20, thermocycling +NaOH, 9-UDMA/bis-EMA/TEGDMA 40/40/20, 5d water + NaOH.

	1	2	3	4	5	6	7	8	9
1	-	0.016656	0.037228	1.000000	0.007118	0.677023	1.000000	0.000265	0.178237
2		-	1.000000	0.001060	1.000000	1.000000	0.012059	1.000000	1.000000
3			-	0.002729	1.000000	1.000000	0.027433	1.000000	1.000000
4				-	0.000393	0.089330	1.000000	0.000009	0.017621
5					-	1.000000	0.005063	1.000000	1.000000
6						-	0.536834	1.000000	1.000000
7							-	0.000177	0.136340
8								-	1.000000
9									-

Ageing protocols: control-water, 24 h, 37 °C; thermocycling + NaOH-water, 7500 thermo cycles, 5/55 °C and 0.1 M NaOH, 7 days, 60 °C; 5d water + NaOH-water, 5 days, 55 °C and 0.1 M NaOH, 7 days.

**Table A3:** Probabilities for *post hoc* testing for three-point bending flexural strength (FS) of tested composites. Tested group: 1-UDMA/bis-GMA/TEGDMA 70/10/20, control; 2-UDMA/bis-GMA/TEGDMA 70/10/20, thermocycling +NaOH, 3-UDMA/bis-GMA/TEGDMA 70/10/20, 5d water + NaOH, 4-UDMA/bis-GMA/TEGDMA 40/40/20, control; 5-UDMA/bis-GMA/TEGDMA 40/40/20, thermocycling +NaOH; 6-UDMA/bis-GMA/TEGDMA 40/40/20, 5d water + NaOH; 7-UDMA/bis-EMA/TEGDMA 40/40/20, control; 8-UDMA/bis-EMA/TEGDMA 40/40/20, thermocycling +NaOH, 9-UDMA/bis-EMA/TEGDMA 40/40/20, 5d water + NaOH.

	1	2	3	4	5	6	7	8	9
1	-	0.099530	0.261666	0.291457	0.000151	0.000591	0.386128	0.022297	0.175759
2		-	0.590310	0.008281	0.019799	0.053406	0.013654	0.501282	0.762280
3			-	0.032130	0.004781	0.014857	0.049649	0.228278	0.813093
4				-	0.000004	0.000017	0.848766	0.001205	0.018123
5					-	0.671329	0.000008	0.090340	0.009106
6						-	0.000034	0.199860	0.026653
7							-	0.002130	0.028840
8								-	0.330947
9									-

Ageing protocols: control-water, 24 h, 37 °C; thermocycling + NaOH-water, 7500 thermo cycles, 5/55 °C and 0.1 M NaOH, 7 days, 60 °C; 5d water + NaOH-water, 5 days, 55 °C and 0.1 M NaOH, 7 days.

**Table A4:** Probabilities for *post hoc* testing for diametral tensile strength (DTS) of tested composites. Tested group: 1-UDMA/bis-GMA/TEGDMA 70/10/20, control; 2-UDMA/bis-GMA/TEGDMA 70/10/20, thermocycling +NaOH, 3-UDMA/bis-GMA/TEGDMA 70/10/20, 5d water + NaOH, 4-UDMA/bis-GMA/TEGDMA 40/40/20, control; 5-UDMA/bis-GMA/TEGDMA 40/40/20, thermocycling +NaOH; 6-UDMA/bis-GMA/TEGDMA 40/40/20, 5d water + NaOH; 7-UDMA/bis-EMA/TEGDMA 40/40/20, control; 8-UDMA/bis-EMA/TEGDMA 40/40/20, thermocycling +NaOH, 9-UDMA/bis-EMA/TEGDMA 40/40/20, 5d water + NaOH.

	1	2	3	4	5	6	7	8	9
1	-	0.347646	0.184878	0.277043	0.000159	0.000018	0.279639	0.013484	0.443427
2		-	0.695213	0.881230	0.003286	0.000494	0.885909	0.116721	0.861824
3			-	0.808370	0.009948	0.001722	0.803782	0.236240	0.571775
4				-	0.005070	0.000803	0.995269	0.154816	0.746411
5					-	0.544665	0.004985	0.150462	0.001949
6						-	0.000788	0.042813	0.000276
7							-	0.153139	0.750901
8								-	0.082238
9									-

Ageing protocols: control-water, 24 h, 37 °C; thermocycling + NaOH-water, 7500 thermo cycles, 5/55 °C and 0.1 M NaOH, 7 days, 60 °C; 5d water + NaOH-water, 5 days, 55 °C and 0.1 M NaOH, 7 days.

**Table A5:** Probabilities for multiple comparison testing for Vickers hardness (HV) of tested composites. Tested group: 1-UDMA/bis-GMA/TEGDMA 70/10/20, control; 2-UDMA/bis-GMA/TEGDMA 70/10/20, thermocycling +NaOH, 3-UDMA/bis-GMA/TEGDMA 70/10/20, 5d water + NaOH, 4-UDMA/bis-GMA/TEGDMA 40/40/20, control; 5-UDMA/bis-GMA/TEGDMA 40/40/20, thermocycling +NaOH; 6-UDMA/bis-GMA/TEGDMA 40/40/20, 5d water + NaOH; 7-UDMA/bis-EMA/TEGDMA 40/40/20, control; 8-UDMA/bis-EMA/TEGDMA 40/40/20, thermocycling +NaOH, 9-UDMA/bis-EMA/TEGDMA 40/40/20, 5d water + NaOH.

	**1**	**2**	**3**	**4**	**5**	**6**	**7**	**8**	**9**
1	-	0.037895	1.000000	1.000000	0.000494	0.000664	1.000000	0.039262	0.110577
2		-	1.000000	0.005379	1.000000	1.000000	0.003224	1.000000	1.000000
3			-	1.000000	0.140756	0.172765	1.000000	1.000000	1.000000
4				-	0.000041	0.000057	1.000000	0.005601	0.018292
5					-	1.000000	0.000022	1.000000	1.000000
6						-	0.000031	1.000000	1.000000
7							-	0.003361	0.011382
8								-	1.000000
9									-

Ageing protocols: control-water, 24 h, 37 °C; thermocycling + NaOH-water, 7500 thermo cycles, 5/55 °C and 0.1 M NaOH, 7 days, 60 °C; 5d water + NaOH-water, 5 days, 55 °C and 0.1 M NaOH, 7 days.

**Table A6:** Results of dispersion and polar components calculations for UDMA/bis-GMA/TEGDMA 70/10/20, UDMA/bis-GMA/TEGDMA 40/40/20 and UDMA/bis-EMA/TEGDMA 40/40/20.

	Dispersion Component [mJ/m^2^]	SD	Polar Component [mJ/m^2^]	SD
UDMA/bis-GMA/TEGDMA 70/10/20	32	5	10	3
UDMA/bis-GMA/TEGDMA 40/40/20	35	3	7	2
UDMA/bis-EMA/TEGDMA 40/40/20	43	2	2	1
